# Attitudes and treatment practice of general practitioners towards patients with obesity in primary care

**DOI:** 10.1186/s12875-020-01239-1

**Published:** 2020-08-17

**Authors:** Maria Schwenke, Melanie Luppa, Alexander Pabst, Franziska D. Welzel, Margrit Löbner, Claudia Luck-Sikorski, Anette Kersting, Matthias Blüher, Steffi G. Riedel-Heller

**Affiliations:** 1 Institute of Social Medicine, Occupational Health and Public Health, Medical Faculty, University of Leipzig, Philipp-Rosenthal-Straße 55, 04103 Leipzig, Germany; 2 Integrated Research and Treatment Centre (IFB) Adiposity Diseases, Leipzig University Medical Center, Leipzig, Germany; 3 SRH University of Applied Health Sciences, Gera, Germany; 4 Clinic for Psychosomatic Medicine and Psychotherapy, University Hospital Leipzig, University of Leipzig, Leipzig, Germany; 5 Institute of General Medicine, University of Leipzig, Leipzig, Germany

**Keywords:** Obesity, Overweight, Primary care, Family practice, Stigmatization

## Abstract

**Background:**

Obesity is one of the most common and relevant health problems in need of urgent action in Germany. General practitioners (GPs) are the initial contact and thus one of the most important starting points for the successful treatment of overweight and obesity. The aim of the study was to assess the treatment practice and attitudes towards patients with obesity in primary health care in Germany.

**Methods:**

Analyses were based on baseline data of the INTERACT trial of 47 GPs in central Germany. Stigmatizing attitudes were identified using the Fat Phobia Scale (FPS). In addition, questionnaires including sociodemographic information, attribution of causes of obesity, referral behavior and clinical activities were completed. Statistical investigations include descriptive analysis, principal component analysis, inference statistics and linear regression models.

**Results:**

GPs rated the quality of medical care for patients with obesity in Germany as below average. The FPS score revealed a value of 3.70, showing that GPs’ attitudes towards patients with obesity are stigmatizing. Younger GP age, male gender and a lower number of referrals to specialists were associated with higher levels of stigmatizing attitudes.

**Conclusion:**

Weight-related stigmatization has an impact on medical treatment. Obesity management guides would help to increase knowledge and reduce weight-related stigmatization in primary care, thereby improving medical care for obese and overweight patients.

## Background

For decades, the prevalence of overweight and obesity has continued to increase [1]. About one-quarter of the German adult population (24% of women, 23% of men) are obese, as defined as having a body mass index (BMI) over 30 kg/m^2^ [2]. Not only is obesity highly prevalent, it is associated with a wide range of other poor health conditions, making intervention is this area highly relevant to improving public health. Further, primary care physicians are at the forefront of the management of obesity in primary care and are crucial to improving outcomes [3]. The chronic nature of obesity calls for a long-term, interdisciplinary health care approach [4]. General practitioners (GPs) are the initial contact for patients’ health care and play a key role in the successful treatment of overweight and obesity [5]. Unfortunately, physicians acknowledge that they lack confidence in managing obesity [6]. Furthermore, they face challenges in weight counseling such as a lack of time and resources. Further, their own negative attitudes towards patients with obesity and a lack of self-efficacy are also relevant [6–8].

It is important to identify current barriers to the weight management of patients in primary care in order to improve their treatment. According to a recent review of obesity management in primary care, Germany has not conducted many surveys on managing obesity, as compared to other countries, in particular the USA [9]. Consistent findings have been reported with regard to stigmatizing attitudes in health care [10]. There are difficulties in making inferences from one country to another, in part, because of sociocultural and structural differences in health care systems. There have been some studies in Germany about stigmatizing attitudes in the general population [11, 12] and health care providers [13], as well as surveys about treatment of obesity [14–16]. However, studies examining the link between attitudes and treatment practice in primary health care are rare.

In this study, we aim to investigate the attitudes, attribution of causes, knowledge and referral and counseling behavior of GPs in central Germany.

## Methods

### Study design and sample

This cross-sectional study used baseline data from the study, “Five As counseling in weight management of patients with obesity in primary care: A cluster-randomized controlled trial (INTERACT)” [17]. The 5As counseling in weight management consists of the following five modules: ASK (recommendations for discussing weight with the patient), ASSESS (assessing health status, comorbidities and causes of weight gain), ADVISE (advising on the health benefits of treatment and available treatment options), AGREE (agreeing on weight loss expectations, treatment plan and treatment goals) and ASSIST (assisting the patient in the continuous process of weight management). Successful weight management is conceptualized as improved overall health and well-being. The design of the INTERACT trial has been reported in detail elsewhere [17, 18].

GPs were recruited between January and May 2016 from an established primary care physician network in central Germany via the Institute of Social Medicine, Occupational Health and Public Health at the University of Leipzig (ISAP). In total, 262 practices were contacted by postal mail and invited to participate. Of these, *n* = 203 did not respond, *n* = 5 declined study participation and *n* = 4 practices were ineligible because the recruitment process had already been completed. The final sample consisted of 47 GPs, none of whom worked together in the same practice.

GPs were asked to indicate how many patients they referred to other medical specialists for help with weight management within the last year and to which specialist patients were referred (e.g. nutrition counseling, specialized treatment center for obesity, bariatric surgery, psychotherapists).

### Instruments

Following recruitment, GPs were asked via standardized, self-rated questionnaires about sociodemographic variables including age, sex, weight/height and work experience. Additionally, the questionnaires contained closed questions on referral behavior (e.g. nutrition counseling, specialized treatment center for obesity, bariatric surgery, psychotherapists), counseling behavior, knowledge about obesity, and attitudes towards obesity and obesity management. Subjective knowledge about obesity was assessed on a 5-point Likert scale ranging from no knowledge to excellent knowledge. In addition, GPs rated the medical care of patients with obesity from poor (score 0) to excellent (score 100).

Stigmatizing attitudes towards obesity were assessed by using the German adaption of the short form of the Fat Phobia Scale (FPS) [12, 19]*.* The FPS contains 14 opposite pairs of adjectives on a scale from 1 (favorable characteristics, e.g. “has will power”, “attractive”) to 5 (unfavorable characteristics, e. g. “no will power”, “unattractive”). A mean FPS score was calculated with higher scores indicating higher negative attribution towards obesity.

GPs were also asked to rate possible causes of obesity from 1 (not important at all) to 5 (highly important) from a list of 10 potential obesity causes: lack of willpower, frequent stress, high caloric intake, lack of physical exercise, hormonal or genetic factors, other somatic disorders, social environment, oversupply of food, lack of knowledge about nutrition and exercise, and insufficient education.

### Statistical analyses

The statistical analyses were performed using SPSS Statistics 24.0 (Statistical Package for Social Science Inc., IBM®, Chicago, IL). Statistical procedures included descriptive analyses of GP characteristics, referral and counseling behavior, and knowledge and attitudes regarding obesity.

To aggregate the causes of obesity, a principal component analysis (PCA) with varimax rotation was conducted. Tests of multicollinearity (Bartlett test of sphericity with *p*-value < 0.05) and sampling adequacy (Kaiser-Meyer-Olkin-criterion (KMO) ≥ 0.50) showed a good fit. The number of components was determined by using Kaiser criterion (eigenvalue > 1). Three variables with factor loadings below 0.6 were excluded. PCA with eigenvalue criterion identified three components: internal causes (lack of willpower, high caloric intake, lack of exercise, somatic disorders), external causes (social environment, oversupply of food) and knowledge (lack of knowledge, insufficient education). For each component, a mean score was calculated.

Associations between stigmatizing attitudes (FPS score) and GP characteristics, treatment activities and causes of obesity (as obtained from PCA) were assessed with linear regression models.

Univariate models were calculated, as were multivariate hierarchical regression models. Model 1 of the hierarchical modelling approach explored the association between stigmatizing attitudes and age and sex of the GP. Model 2 additionally included the number of referred patients within 1 year to assess the influence of treatment behavior. Model 3 added the subjective attitudes of causes of obesity, summarized by PCA, to explain negative attitudes towards obesity. Work experience was not included in the models due to multicollinearity with age (*r* = 0.90, *p < 0.001*). For all statistical analyses, the level of statistical significance was assumed at *p*-value ≤0.05.

## Results

### Characteristics

Sociodemographic characteristics of the GPs are shown in Table 1. The majority of the 47 recruited GPs were female (59.6%). BMI ranged from 18.14 kg/m^2^ to 31.80 kg/m^2^. Almost two-thirds of the GPs had normal weight (BMI 18.6–24.9 kg/m^2^) and almost one-fourth had overweight (BMI 25.0–29.9 kg/m^2^). Three of the 49 GPs (6.4%) had obesity class I (BMI 30.0–34.9 kg/m^2^) and one person (2.1%) had underweight (BMI < 18.5 kg/m^2^).

**Table 1:** Sample characteristics (*n* = 47)

Characteristics	Values
Age in years, Mean (SD)	48.70 (8.69)
Sex, n (%)	
Male	19 (40.4)
Female	28 (59.6)
Work experience^a^ in years, Mean (SD)	20.57 (9.89)
BMI^b^ in kg/m^2^, Mean (SD)	23.96 (2.95)

^a^Missing data for *n* = 1 (2.1%) participant, ^b^Missing data for *n* = 2 (4.2%) participants, *BMI* body mass index

### GP referral and counseling behavior

GPs reported the mean percentage of patients with obesity in their practices per month as almost one-third (28.5%). They further reported the mean number of patients who were referred to specialists by their GPs in the last 12 months at 28. Most were referred to dietitians, followed by obesity treatment centers. Within a medical consultation, the risks of excessive body weight were addressed regularly by 87% of the GPs. Frequently performed clinical activities associated with patients with obesity include: check-up and investigation of comorbidities, BMI assessment and advice on physical activity and other behavioral changes. Less frequently, GPs gave detailed advice on diet and measured waist circumference (see Table 2).

**Table 2:** Referral and counseling behavior (*n* = 47)

Variables	Values
Percent of overweight patients per month, Mean (SD)	28.51 (13.92)
Number of referred patients in the last 12 month^a^, Mean (SD)	28.23 (37.62)
‘To how many patients have you given a recommendation for bariatric surgery in the last 5 years?’, Mean (SD)	4.89 (8.25)
Frequency of conducted clinical activities, n (%)	
Assessment of BMI	
Frequently	41 (87.2)
Rarely to never	6 (12.7)
Waist circumference measurement	
Frequently	22 (46.8)
Rarely to never	25 (53.2)
Check-up / treatment of comorbidities	
Frequently	45 (95.7)
Rarely to never	2 (4.3)
Comprehensive counseling for behavior change	
Frequently	42 (89.4)
Rarely to never	5 (10.6)
Comprehensive counseling on diet^a^	
Frequently	27 (57.4)
Rarely to never	19 (40.4)
Comprehensive counseling on physical activity	
Frequently	46 (97.9)
Rarely to never	1 (2.1)

^a^Missing data for *n* = 1 (2.1%) participant

### Knowledge

More than three-quarters of GPs rated their knowledge of the treatment of obesity as good to very good (79%). However, knowledge about obesity surgery was mixed: 34% reported their knowledge as good to very good, 40% were familiar with it and 26% knew not much or nothing about obesity surgery. Spearman rank correlation revealed a significant positive correlation between work experience in years and subjective assessment of knowledge about obesity (*r* = 0.356, *p* = 0.015). Only three GPs (6.4%) reported that the topic of obesity had been addressed with reasonable sufficiency in their specialist medical training and two persons (4.3%) said it was addressed with reasonable sufficiency in their educational training. By contrast, about 60% reported that the issue of obesity was either not at all or not sufficiently addressed in educational and medical training. Almost 60% of GPs reported that they would like more training on the treatment of obesity.

### Attitudes and attribution of causes

**Fig. 1:**
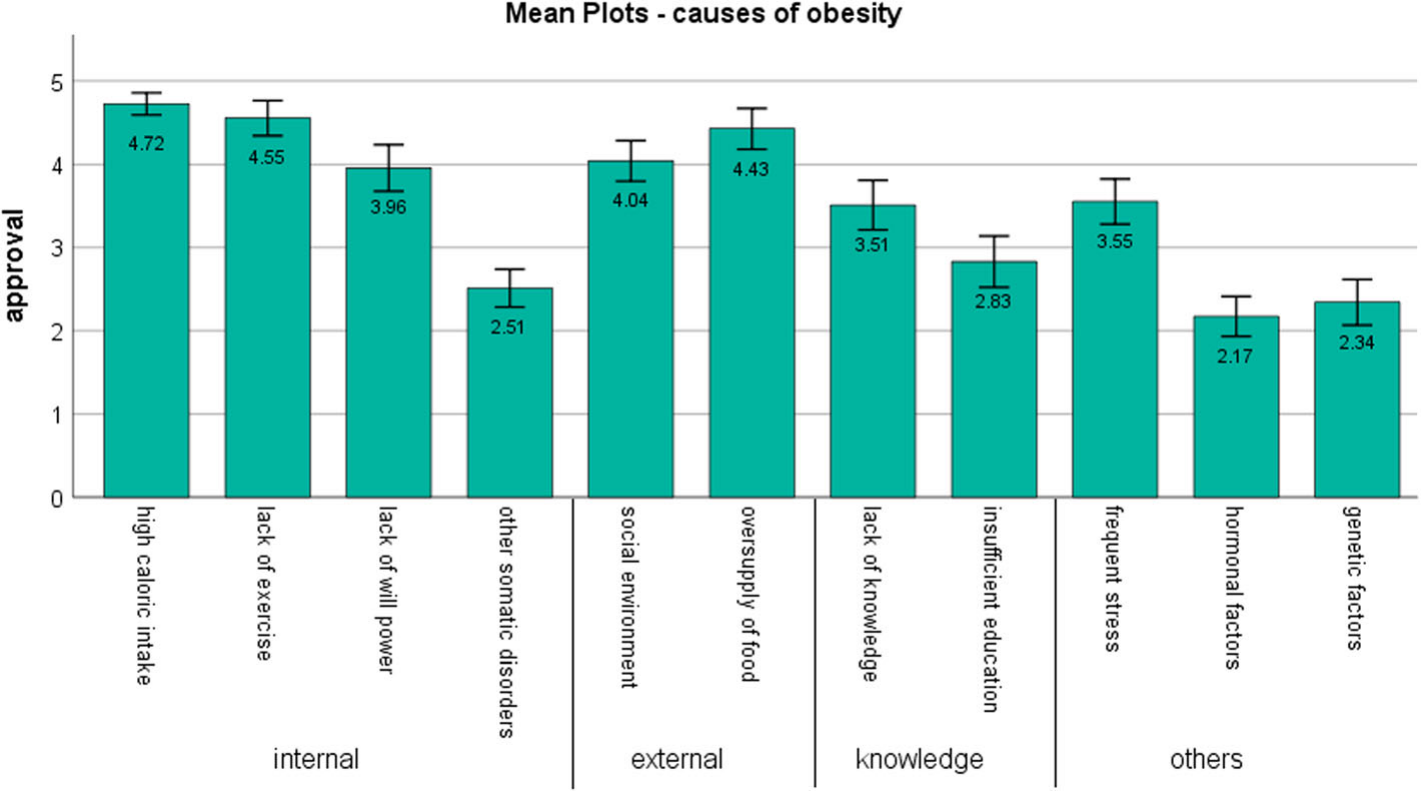
Mean Plots - Attribution to causes of obesity. Notes: display of mean values and error bars (95% confidence interval), summarizing causes of obesity according to results of Principal Component Analysis

When asked to rate the quality of medical care of patients with obesity in Germany, GPs in our study estimated quality of treatment at 47 of 100 (Table 3). Data on the importance of the causes of obesity as rated by the GPs is presented in Fig. 1. The highest scores (median of 5; extremely meaningful) were assigned to “high caloric intake”, “lack of physical exercise” and “oversupply of food”. In addition, causes regarded as important (median of 4) were “social environments”, “the weakness of will”, “frequent stress” and a “lack of knowledge” about nutrition and exercise. The cause “insufficient education” was rated across all response categories (median of 3). However, physiological causes like hormonal or genetic factors and other somatic disorders were rated as less important (median of 2). PCA with eigenvalue criterion summarized the causes into three components: internal causes, external causes and knowledge (Table 3).

**Table 3:** Attitudes of GPs about obesity (n = 47)

Variables	Values
Estimation of quality of medical care for patients with obesity (scale: 0–100)^a^	46.60 (18.15) Range 10–100
Fat Phobia Scale FPS (scale: 1–5)^a^, Mean (SD)	3.70 (0.36) Range 3.00–4.29
Attribution of causes (scale: 1–5)^a^	
Internal causes; Mean (SD)	3.94 (0.54)
External causes; Mean (SD)	4.23 (0.67)
Knowledge; Mean (SD)	3.17 (0.92)

^a^higher values indicate higher agreement

The Fat Phobia Scale was used to assess stigmatizing attitudes; the mean score of the 5-point rating scale was 3.70 (Table 3). Values over 4.0, which indicated a very high approval, were found for items: “likes food” (4.51 ± 0.72) and “overeats” (4.28 ± 0.97). Just behind it with a value of 3.94 (SD = 0.53) was the attribution “no willpower”. The lowest value was found for the attribution “poor self-control” (3.11 ± 0.75). A value under 2.5 would indicate neutral or positives attitudes. Indeed, in the sample no attribution was scored below 3.00.

Univariate linear regression models of determinants on mean FPS score revealed a significant age effect (*b* = − 0.016, *p* = 0.005). Younger GPs reported higher FPS scores which indicated more negative attitudes towards patients with obesity. All other variables on sociodemographics, treatment, knowledge and attribution of causes were not univariately associated with stigmatizing attitudes. Results of the multivariate regression analyses are shown in Table 4. In Model 1, both age (*b* = − 0.017, *p* = 0.004) and gender (*b* = − 0.207, *p* = 0.039) were independently associated with stigmatization, with older age and female gender showing less negative views towards patients with obesity. Model 2 revealed that, adjusting for sociodemographics, GPs with higher numbers of referred patients in the last 12-months displayed slightly lower levels of stigmatizing attitudes (*b* = − 0.003, *p* = 0.035), although the association was borderline significant. Model 3 showed that the attribution of causes does not contribute to the elucidation of stigmatizing attitudes when adjusting for sociodemographics and patient referral (Table 4).

**Table 4:** Linear regression models of the Fat Phobia Scale

	Variable	Model 1			Model 2			Model 3		
		Beta-coefficient [95% CI]	Stand. Beta	***p***-value	Beta-coefficient [95% CI]	Stand. Beta	***p***-value	Beta-coefficient [95% CI]	Stand. Beta	***p***-value
Sociodemographic	Age	−0.017 [**−** 0.028, − 0.006]	−0.409	0.004	−0.018 [− 0.028, − 0.007]	−0.427	0.002	−0.018 [− 0.029, − 0.007]	−0.441	0.002
	Sex (ref. male)	−0.207 [− 0.403, − 0.011]	−0.285	0.039	−0.251 [− 0.444, − 0.059]	−0.346	0.012	−0.253 [− 0.445, − 0.049]	−0.340	0.016
Treatment	Number of referred patients				−0.003 [− 0.005, 0.000]	−0.285	0.035	−0.003 [− 0.005, 0.000]	−0.275	0.055
Attribution of causes	Internal							0.086 [−0.097, 0.269]	0.128	0.348
	Knowledge							−0.016 [−0.121, 0.089]	−0.041	0.761
	External							−0.053 [−0.204, 0.099]	− 0.097	0.487

*CI* confidence interval

## Discussion

The aim of the present study was to describe the treatment practice of obesity in primary care in central Germany and to investigate the associations with stigmatizing attitudes.

The reported mean percentage of patients with overweight was 28.5 each month. This appears to be low considering that the national rate of overweight is 67% of men and 53% of women [2]. One cause could be a low recognition rate. This notion is supported by Bramlage et al., in which only 20–30% of patients with overweight were recognized as being overweight by GPs [14]. Results of a French [20] and a Hungarian study [21] showed that most GPs underestimated the prevalence of overweight. GPs in our study may thus consider overweight to be normal, because the majority of the German population is overweighed and therefore it is not explicitly diagnosed [14, 22]. However, early recognition of weight problems is important for prevention of obesity [5, 20].

In our study, all GPs showed moderately to highly stigmatizing attitudes towards patients with obesity. The mean FPS score (3.70) is slightly higher than that estimated in the German general population (FPS = 3.62) [12]. Also, health care professionals in Germany showed comparable stigmatizing attitudes towards female patients with obesity (FPS = 3.59) [13]. A previous study from Germany identified similar stigmatizing attitudes in medical students (FPS = 3.65) [23]. Most GPs in this study estimated that the topic obesity was addressed “not at all” or “hardly sufficiently” in medical training or education. In addition, the majority would like to have more training on the topic of treating obesity. Therefore, the issue of obesity should be fully addressed during medical education and be refreshed by regular trainings during clinical activity to reduce negative attitudes towards overweight and obesity.

In the present analysis, we have observed associations between stigmatizing attitudes and GPs characteristics. First, higher age was associated with lower stigmatizing attitudes. This is in line with results of the general population in Germany [12]. In contrast, a German study of health care professionals reported different results; they found increased age to be associated with more stigmatizing attitudes [13]. However, the study also showed that with increasing work experience the FPS values decreased [13], which is also reflected in our results. Similarly, a study of professionals treating eating disorders also showed an effect between more years of professional experience and less negative attitudes [24]. Furthermore, a positive professional experience was correlated with lower explicit weight bias [25] and directly working with people with obesity seems to counteract the weight stigmatization [26]. Further, we observed a gender-related effect on weight stigmatization. Female physicians had less negative attitudes than males, which confirms previous research [27]. It may be because women are more likely to be affected by weight-related stigmatization and are therefore more sensitive to prejudice and discrimination [28]. We also found an association between the GPs treatment practice and the stigmatization of patients with obesity. GPs with a higher number of referrals endorsed less stigmatizing attitudes. According to our findings, GPs mostly refer their patients to dietitians or obesity treatment centers. This is consistent with other reports that lifestyle changes were assessed to be the most effective available intervention option [6, 29]. The referral intention of GPs depends on several factors, as shown by a qualitative study from Australia [29]. Attitudes and perceived experience were the most influencing factors on GPs referral intent, which is supported by our results. Knowledge and stigmatizing attitudes towards treatment options also seems to have an influence on referral behavior, as shown by a German study on weight loss surgery [16]. In addition, the availability of resources, such as the possibility for direct contact between GPs and referral providers and the location of the referral service, influences GP decisions [6, 22, 29]. It is likely that the absence of referral options leads to difficulties in obesity treatment and this may result in frustration and stigmatization of the patient. GP attitudes may be influenced by limited case experience and a lack of knowledge about positive treatment outcomes [6]. For these reasons, close cooperation with potential referrers and health care providers seems to be important for optimal obesity treatment and for counteracting stigmatizing attitudes. It should be mentioned that the reimbursement system of the health insurances in Germany does not cover obesity as single disease, rather only illnesses which occur subsequently or are associated with obesity [5]. This creates structural barriers in GPs’ treatment and referral options for obesity therapy.

We did not find a significant association between the attribution of causes of obesity and stigmatizing attitudes of GPs, which could have resulted from a lack of statistical power due to the small sample. Previous research has shown that a higher attribution to biomedical causes is associated with more positive attitudes towards women with obesity, while an attribution to personal responsibility leads to more negative views [11, 13]. Another study showed that physiological causes were negatively associated with FPS, whereas behavioral causes had a positive association with stigmatizing attitudes [24, 27]. The present analysis also found the highest response category to causes of obesity were personal behavioral factors such as high caloric intake and low physical activity; rather low was the endorsement of physiological and genetic causes. It is possible that GPs believe in a victim blaming approach to the weight problem [30–32]. This implies that health care professionals believe that obesity is controllable and that important causes of obesity are within the patient’s control – like an unbalanced energy intake [13, 23, 30]. In contrast, the general public believe more in the medical causes of obesity and that the GPs are helpful in weight management [30, 33]. This disagreement could affect the patient-provider relationship [20]. Results based on the present INTERACT study suggest that the patient-GP interaction measured with the instrument, “Patient assessment of chronic illness care (PACIC-5A)” is rated as low from patient perspective [18]. However, a good patient-GP relationship and high quality weight consultation are important for patient care [34, 35].

Some limitations of this study should be mentioned. GPs were contacted and asked to provide information on treatment of obesity in primary care. This could have created a pre-selection bias of GPs who were already concerned with the topic. Compared to the German population of GPs in primary care, our sample was considerably younger (48.7 vs. 55.4 years [36]) and more often female (59.6% vs. 43.9% [35]). Furthermore, we collected data by questionnaires not controlling for social desirability or validity of the answers. Additionally, the use of standardized interviews limits the collection of more detailed data on treatment behavior, attitudes and knowledge of GPs as possible with qualitative methods. Furthermore, in the list of potential causes of obesity medications were not included which are also known to cause weight gain, e. g. anti-psychotic medications. Moreover, the small sample size impacts the results because of lack of statistical power, especially, as related to the lack of association between attribution of causes and stigmatizing attitudes. Generalizability should also be seen as restricted to GPs in central Germany who are concerned with the topic of obesity.

## Conclusion

Despite the high prevalence rates of overweight and obesity in the population, weight problems do not seem to be regularly diagnosed in general practice. However, early recognition and treatment of overweight and obesity are important to avoid sequelae. Further training for physicians is needed on the complexity of the etiology of obesity including obesity management programs to improve treatment and reduce stigmatizing attitudes in GPs. For this purpose, the 5A approach for obesity management is recommended as a guide for health care professionals to optimize obesity care and reduce weight-related stigmatization [17, 37]. Obesity management should include physicians as gatekeepers with nutrition experts, nurses, psychologists and physiotherapists providing additional services. Primary care for patients with obesity should become more structured, interdisciplinary, and innovative to optimize patient care in Germany [5, 38]. For future research, it would be interesting to investigate other health care professionals as well. Information on the treatment practice and attitudes of the stakeholders could provide a pathway for improving cooperation.

## Data Availability

The datasets generated and analyzed during the current study are not publicly available due to ethical restrictions and patient confidentiality but are available from the corresponding author on reasonable request. Aggregated data are provided in the paper tables.

## Abbreviations

BMI: Body Mass Index
CI: Confidence Interval
FPS: Fat Phobia Scale
FU: Follow-Up
GP: General Practitioners
IFB: Integrated Research and Treatment Centre
ISAP: Institute of Social Medicine, Occupational Health and Public Health of the University of Leipzig
KMO: Kaiser-Meyer-Olkin-Criterion
PACIC: Patient Assessment of Chronic Illness Care
PCA: Principal Component Analysis
